# Regulation of dendritic spine length in corticopontine layer V pyramidal neurons by autism risk gene β3 integrin

**DOI:** 10.1186/s13041-023-01031-z

**Published:** 2023-06-09

**Authors:** Lucia Celora, Fanny Jaudon, Carmela Vitale, Lorenzo A. Cingolani

**Affiliations:** 1 Department of Life Sciences, University of Trieste, Trieste, 34127 Italy; 2 IRCCS Ospedale Policlinico San Martino, Genoa, 16132 Italy; 3 Center for Synaptic Neuroscience and Technology (NSYN), Fondazione Istituto Italiano di Tecnologia (IIT), Genoa, 16132 Italy

**Keywords:** *Itgb3*, Medial prefrontal cortex, ASD, Dendritic spines, Retrograde labelling

## Abstract

The relationship between autism spectrum disorder (ASD) and dendritic spine abnormalities is well known, but it is unclear whether the deficits relate to specific neuron types and brain regions most relevant to ASD. Recent genetic studies have identified a convergence of ASD risk genes in deep layer pyramidal neurons of the prefrontal cortex. Here, we use retrograde recombinant adeno-associated viruses to label specifically two major layer V pyramidal neuron types of the medial prefrontal cortex: the commissural neurons, which put the two cerebral hemispheres in direct communication, and the corticopontine neurons, which transmit information outside the cortex. We compare the basal dendritic spines on commissural and corticopontine neurons in WT and KO mice for the ASD risk gene *Itgb3*, which encodes for the cell adhesion molecule β3 integrin selectively enriched in layer V pyramidal neurons. Regardless of the genotype, corticopontine neurons had a higher ratio of stubby to mushroom spines than commissural neurons. β3 integrin affected selectively spine length in corticopontine neurons. Ablation of β3 integrin resulted in corticopontine neurons lacking long (> 2 μm) thin dendritic spines. These findings suggest that a deficiency in β3 integrin expression compromises specifically immature spines on corticopontine neurons, thereby reducing the cortical territory they can sample. Because corticopontine neurons receive extensive local and long-range excitatory inputs before relaying information outside the cortex, specific alterations in dendritic spines of corticopontine neurons may compromise the computational output of the full cortex, thereby contributing to ASD pathophysiology.

**Supplementary Information:**

The online version contains supplementary material available at 10.1186/s13041-023-01031-z.

## Main text

**Fig. 1:**
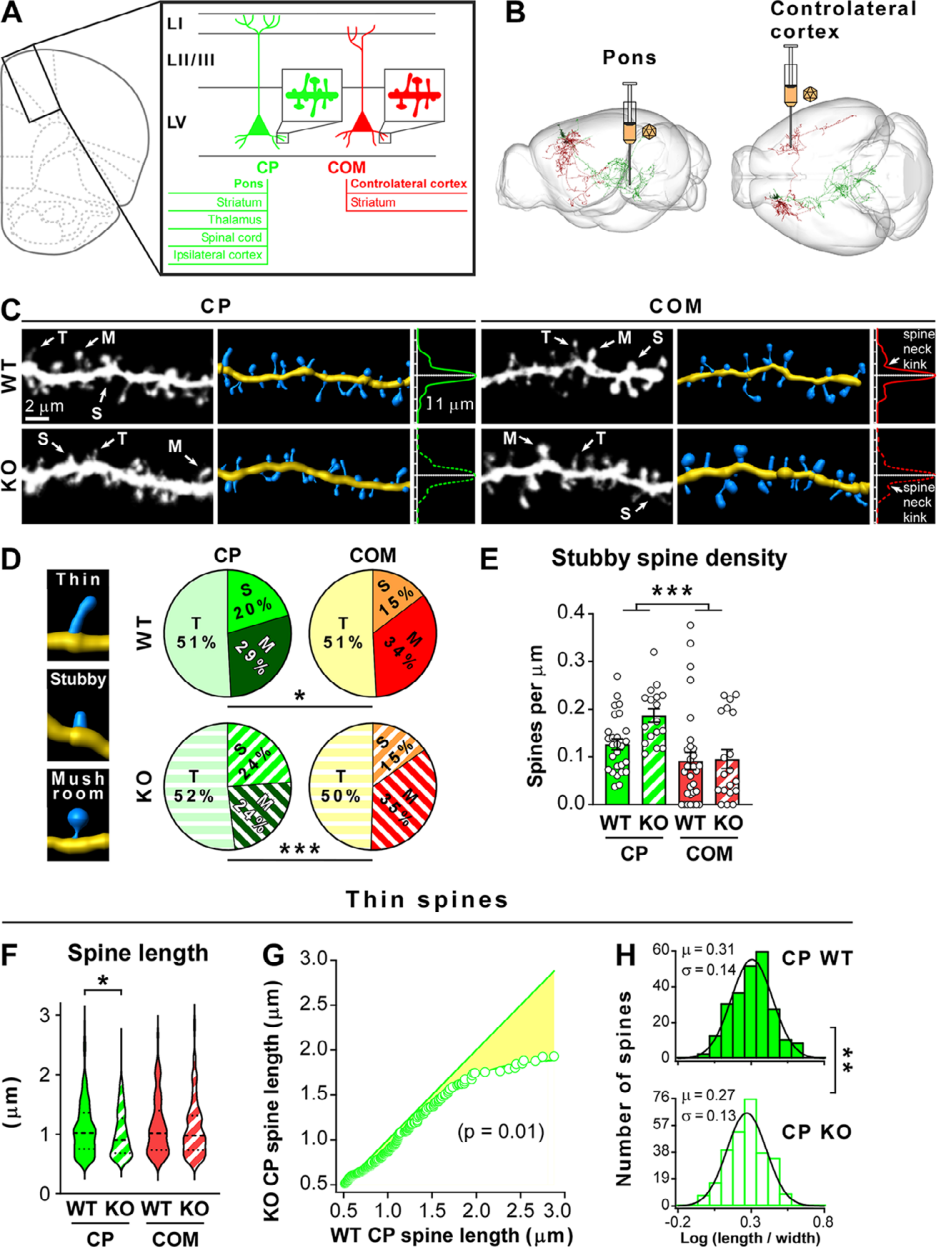
Corticopontine but not commissural layer V pyramidal neurons exhibit shorter thin dendritic spines in *Itgb3* KO mice **(A)** Major outputs of LV mPFC pyramidal neurons include the pons for pyramidal tract neurons (corticopontine [CP] neurons; green) and the contralateral cortex for intratelencephalic neurons (commissural [COM] neurons; red). **(B)** The retrograde rAAV AAVrg-hSyn-EGFP was injected in the pons or contralateral mPFC to label CP or COM neurons, respectively. One CP (red) and one COM neuron (green) are shown in sagittal and horizontal views (images generated using the MouseLight interface; ID AA0261 and AA0656, https://www.janelia.org/project-team/mouselight/neuronbrowser). **(C)** Representative images and Imaris 3D rendering of basal dendrites from CP WT, CP *Itgb3* KO, COM WT and COM *Itgb3* KO LV pyramidal neurons. Arrows point to representative thin (T), mushroom (M) and stubby (S) spines. Loss of *Itgb3* reduces spine length in CP neurons. Intensity profiles of the same dendrites after straightening reveal a ‘spine neck kink’ only in the COM WT and COM KO conditions, suggesting that COM neurons (WT and KO alike) exhibit spines with thinner spine necks than CP neurons (WT and KO alike). **(D)** Left: sample Imaris 3D rendering of thin, stubby and mushroom spines. Right: pie chart of spine type distribution (*p = 0.03; ***p = 0.0003; Chi-square test; n = 482, 532, 476 and 317 spines for CP WT, CP *Itgb3* KO, COM WT and COM *Itgb3* KO, respectively). **(E)** Density of stubby spines (two-way ANOVA; genotype effect: F (1, 90) = 3.548, p = 0.0628; neuron type effect: F (1, 90) = 13.45, ***p = 0.0004; genotype x neuron type interaction: F (1, 90) = 2.670, p = 0.1057; n = 27, 17, 31 and 19 dendritic stretches for CP WT, CP KO, COM WT and COM KO, respectively). **(F)** Violin plot for the length of thin spines (*p = 0.01, non-parametric Kruskal-Wallis ANOVA followed by Benjamini, Krieger and Yekutieli post-test, which corrects for multiple comparisons by controlling the false discovery rate; n = 245, 276, 242 and 157 thin spines for CP WT, CP *Itgb3* KO, COM WT and COM *Itgb3* KO, respectively). In each violin plot, the thick dotted line and the two thin dotted lines indicate the median and the quartiles, respectively. **(G)** Thin spines of CP *Itgb3* KO neurons were ranked according to their length, resampled to match the number of thin spines of CP WT neurons and plotted against the ranked thin spines of CP WT neurons (straight line: CP WT vs. CP WT; open circles: CP *Itgb3* KO vs. CP WT; line through open circles is a sigmoid fit; p = 0.01; Kolmogorov-Smirnov test; n = 245 and 276 thin spines for CP WT and CP *Itgb3* KO, respectively; full data set in supplemental Fig. 2). **(H)** Histogram of the ratio between length and head width for thin spines of CP WT and CP *Itgb3* KO neurons on a logarithmic scale (Log (length / width)) reveals a log-normal distribution for this morphological parameter (**p = 0.004 between CP WT and CP *Itgb3* KO, parametric Brown-Forsythe and Welch ANOVA followed by Benjamini, Krieger and Yekutieli post-test; full data set in supplemental Fig. 2). Continuous lines are Gaussian fits with the indicated means (µ) and standard deviations (σ)

While dendritic spine abnormalities are a hallmark of many forms of autism spectrum disorder (ASD; [1]), it is generally not known whether these deficits correlate with brain regions and neuron types most relevant to ASD. Human genetic studies have consistently identified a convergence of ASD risk genes in deep layer pyramidal neurons of the prefrontal cortex [2]. Two major types of pyramidal neurons, with divergent functions, are found intermingled in the deep cortical layer V (LV) of the medial prefrontal cortex (mPFC). Intratelencephalic neurons, whose axons project only within the telencephalon, including the contralateral cortex (commissural [COM] neurons) and pyramidal tract neurons, whose axons remain ipsilateral within the telencephalon and project to distant subcerebral regions, including the pons (corticopontine [CP] neurons; Fig. 1A; [3]). While the dendritic arborization, neuromodulation and electrophysiological properties of COM and CP neurons have been extensively investigated, technical difficulties in differentially labeling them have so far precluded a comparative analysis of their dendritic spines. Likewise, we do not know whether ASD risk genes affect dendritic spines on both types of neurons or only on one of them, thereby skewing how LV cortical circuits integrate synaptic inputs.

Here we used retrograde recombinant adeno-associated viruses (retro-rAAVs; [4] injected into the contralateral cortex or pons to label unambiguously COM or CP neurons, respectively (Fig. 1B). This allowed us to address two questions: First, are density and morphology of basal dendritic spines different between COM and CP neurons? Second, are these spines abnormal in KO mice for the ASD risk gene β3 integrin (*Itgb3*)? We focused on *Itgb3* KO mice because (i) the cell adhesion molecule β3 integrin is enriched in human and mouse LV pyramidal neurons [5, 6], (ii) its association to ASD is supported by both single nucleotide polymorphisms and rare mutations [7], (iii) *Itgb3* KO mice exhibit autism-like behaviors [8] and (iv) members of the integrin family have previously been shown to be important for synaptic plasticity and dendritic spine dynamics [9, 10].

Regardless of the genotype, dendritic spine density was slightly but significantly higher in CP than in COM neurons (Fig. 1C and S1A). This prompted us to investigate whether there were differences in the distribution and density of specific spine subtypes. We therefore classified dendritic spines into three categories (thin, stubby and mushroom), according to morphological criteria (Fig S1E). Compellingly, CP neurons had a higher ratio of stubby to mushroom spines than COM neurons (Fig. 1D), and this was mainly due to a higher density of stubby spines in CP neurons (Fig. 1E and S1C, D). Albeit more pronounced in *Itgb3* KO mice, the differences between neuronal types were present also in WT mice, and were therefore unlikely due to β3 integrin. Notably, the change in the relative distribution between mushroom and stubby spines (which do and do not have visible spine necks, respectively) was also evident in individual dendrites as a spine neck ‘kink’ in the transversal fluorescence profiles of straighten dendrites in COM but not CP neurons (Fig. 1C). No difference was instead detected in the relative percentage or density of thin spines between neuron types or genotypes (Fig. 1C, D and S1B).

The functional role of stubby spines is debated. While they have traditionally been seen as immature spines, recent studies indicate that a large proportion of stubby spines could be a form of potentiated mushroom spines with very short necks [11]. Regardless, our findings suggest that the number of spines with short and large necks, thus having low diffusional coupling between spine head and parental dendrite, is higher in CP than COM neurons.

β3 integrin affected selectively spine length in CP but not COM neurons. Specifically, ablation of β3 integrin shortened overall spine length in CP neurons, with the effect being largely due to a reduction in the length of thin spines (Fig. 1C, F, S2). Notably, CP KO neurons did not uniformly scale down the length of thin dendritic spines but were most deficient in thin spines longer than ~ 2 μm (Fig. 1C, G, S2). Because length and head width of dendritic spines were highly heterogeneous, even within each dendritic spine subtype (Fig S2), we examined the distributions of the ratio between spine length and head width, which were found to be positively skewed. We therefore plotted histograms of the logarithm of the spine length to head width ratio, which revealed log-normal distributions of this morphological parameter across spine subtypes (Fig. 1H, S2). Parametric statistical analyses of the transformed data confirmed the specific effect of β3 integrin on thin spines of CP neurons (Fig. 1H, S2).

Taken together, our findings suggest that a deficiency in the ASD risk gene β3 integrin compromises preferentially immature thin spines on CP neurons. Because these are dynamic spines that explore the area surrounding their parental dendrite before forming stable synaptic contacts [1], loss of β3 integrin may reduce the ability of CP neurons to do so, potentially altering their final choice for synaptic partners. Recent data indicate that β3 integrin may have an early, rather than late, function in dendritogenesis [12]. Likewise, this integrin is specifically required for the initiation of neuronal differentiation in neuroblastoma N2a cells [13]. Thus, β3 integrin could play a similar role in CP neurons by promoting spine elongation, because it generates traction forces at adhesion contacts of thin spines, or by preventing spine retraction, because it contributes to the initial and dynamic contacts between synaptic partners. This is reminiscent of the role played by β1 integrin, which maintains immature spines of primary hippocampal neurons in a highly dynamic state by interacting with the cell adhesion molecule telencephalin [14]. A limitation of our study is that we analyzed only dendritic spines on basal dendrites since retrograde labeling prevented us from determining whether apical dendrites in layers I-III originated exclusively from CP or COM neurons of LV. Long-range excitatory inputs to basal dendrites in LV are biased towards CP or COM neurons: inputs from the contralateral cortex and basolateral amygdala target preferentially CP neurons while those from the ventral hippocampus are biased towards COM neurons. Local connectivity is also largely asymmetric, with COM neurons projecting mostly unidirectionally to CP neurons, which, in turn, convey information outside the cortex [3, 15]. Alterations in dendritic spines specific to CP neurons may therefore compromise the computational output of the full cortex, thereby contributing to ASD pathophysiology.

## Electronic supplementary material

Below is the link to the electronic supplementary material.

Supplementary Material 1

## Data Availability

The datasets generated during and/or analyzed during the current study are available from the corresponding author.

## Abbreviations

ASD: Autism spectrum disorder
COM: Commissural
CP: Corticopontine
LV: Layer V
mPFC: Medial prefrontal cortex
retro-rAAV: Retrograde recombinant adeno-associated virus.

## References

[1] Kasai H, Ziv NE, Okazaki H, Yagishita S, Toyoizumi T (2021). Spine dynamics in the brain, mental disorders and artificial neural networks. Nat Rev Neurosci.

[2] Willsey HR, Willsey AJ, Wang B, State MW (2022). Genomics, convergent neuroscience and progress in understanding autism spectrum disorder. Nat Rev Neurosci.

[3] Baker A, Kalmbach B, Morishima M, Kim J, Juavinett A, Li N (2018). Specialized subpopulations of deep-layer pyramidal neurons in the neocortex: bridging Cellular Properties to Functional Consequences. J Neurosci.

[4] Tervo DG, Hwang BY, Viswanathan S, Gaj T, Lavzin M, Ritola KD (2016). A designer AAV variant permits efficient Retrograde Access to Projection neurons. Neuron.

[5] Belgard TG, Marques AC, Oliver PL, Abaan HO, Sirey TM, Hoerder-Suabedissen A (2011). A transcriptomic atlas of mouse neocortical layers. Neuron.

[6] Willsey AJ, Sanders SJ, Li M, Dong S, Tebbenkamp AT, Muhle RA (2013). Coexpression networks implicate human midfetal deep cortical projection neurons in the pathogenesis of autism. Cell.

[7] Jaudon F, Thalhammer A, Cingolani LA (2021). Integrin adhesion in brain assembly: from molecular structure to neuropsychiatric disorders. Eur J Neurosci.

[8] Carter MD, Shah CR, Muller CL, Crawley JN, Carneiro AM, Veenstra-Vanderweele J (2011). Absence of preference for social novelty and increased grooming in integrin beta3 knockout mice: initial studies and future directions. Autism Res.

[9] Jaudon F, Thalhammer A, Zentilin L, Cingolani LA (2022). CRISPR-mediated activation of autism gene Itgb3 restores cortical network excitability via mGluR5 signaling. Mol Ther Nucleic Acids.

[10] Kerrisk ME, Cingolani LA, Koleske AJ (2014). ECM receptors in neuronal structure, synaptic plasticity, and behavior. Prog Brain Res.

[11] Tonnesen J, Katona G, Rozsa B, Nagerl UV (2014). Spine neck plasticity regulates compartmentalization of synapses. Nat Neurosci.

[12] Swinehart BD, Bland KM, Holley ZL, Lopuch AJ, Casey ZO, Handwerk CJ (2020). Integrin β3 organizes dendritic complexity of cerebral cortical pyramidal neurons along a tangential gradient. Mol Brain.

[13] Riccardi S, Cingolani LA, Jaudon F (2022). CRISPR-Mediated activation of alphaV integrin subtypes promotes neuronal differentiation of Neuroblastoma Neuro2a cells. Front Genome Ed.

[14] Ning L, Tian L, Smirnov S, Vihinen H, Llano O, Vick K (2013). Interactions between ICAM-5 and beta1 integrins regulate neuronal synapse formation. J Cell Sci.

[15] Anastasiades PG, Carter AG (2021). Circuit organization of the rodent medial prefrontal cortex. Trends Neurosci.

