# Supplementary material for: Regulation of dendritic spine length in corticopontine layer V pyramidal neurons by autism risk gene β3 integrin
Source: Mol Brain. 2023 Jun 9;16:49. doi: 10.1186/s13041-023-01031-z (PMC10251541; doi:10.1186/s13041-023-01031-z)
Supplement: Supplementary file 1 — Supplementary Material 1 [file 13041_2023_1031_MOESM1_ESM.pdf]

Supplemental information

Regulation of dendritic spine length in corticopontine layer V pyramidal neurons by autism risk gene β3 integrin

Lucia Celora[1, *], Fanny Jaudon[1, 2, *], Carmela Vitale[3] and Lorenzo A. Cingolani[1, 3, 4]

[1] Department of Life Sciences, University of Trieste, 34127 Trieste, Italy

[2] IRCCS Ospedale Policlinico San Martino, 16132 Genoa, Italy

[3] Center for Synaptic Neuroscience and Technology (NSYN), Fondazione Istituto Italiano di Tecnologia (IIT), 16132 Genoa, Italy

[4] Correspondence: lcingolani@units.it

* These authors contributed equally to this study

Supplemental figures

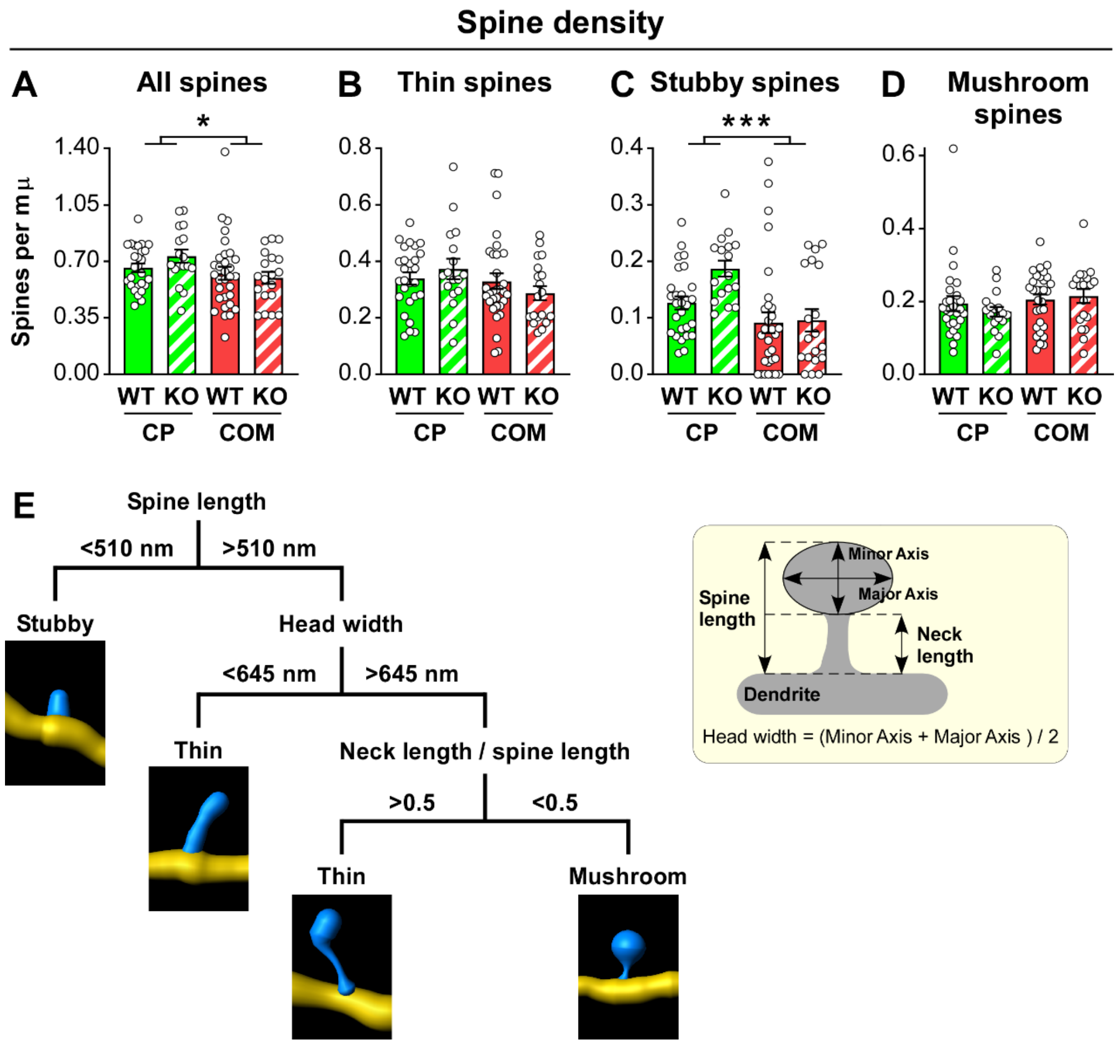

Supplemental Figure 1. Dendritic spine density. (A) Density of all dendritic spines (two-way ANOVA; genotype effect: F (1, 90) = 0.3154, p = 0.5758; neuron type effect: F (1, 90) = 4.746, *p = 0.032; genotype x neuron type interaction: F (1, 90) = 1.685, p = 0.1976; n = 27, 17, 31 and 19 dendritic stretches for CP WT, CP KO, COM WT and COM KO, respectively). **(B)** Density of thin spines (two-way ANOVA; genotype effect: F (1, 90) = 0.02147, p = 0.8838; neuron type effect: F (1, 90) = 2.819, p = 0.0966; genotype x neuron type interaction: F (1, 90) = 1.766, p = 0.1872). **(C)** Density of stubby spines; same data as in panel (E) of Fig 1 (two-way ANOVA; genotype effect: F (1, 90) = 3.548, p = 0.0628; neuron type effect: F (1, 90) = 13.45, ***p = 0.0004; genotype x neuron type interaction: F (1, 90) = 2.670, p = 0.1057). **(D)** Density of mushroom spines (two-way ANOVA; genotype effect: F (1, 90) = 0.1371, p = 0.7120; neuron type effect: F (1, 90) = 2.269, p = 0.1355; genotype x neuron type interaction: F (1, 90) = 0.7915, p = 0.3760). **(E)** Morphological criteria used for the classification of dendritic spines into stubby, thin and mushroom types.

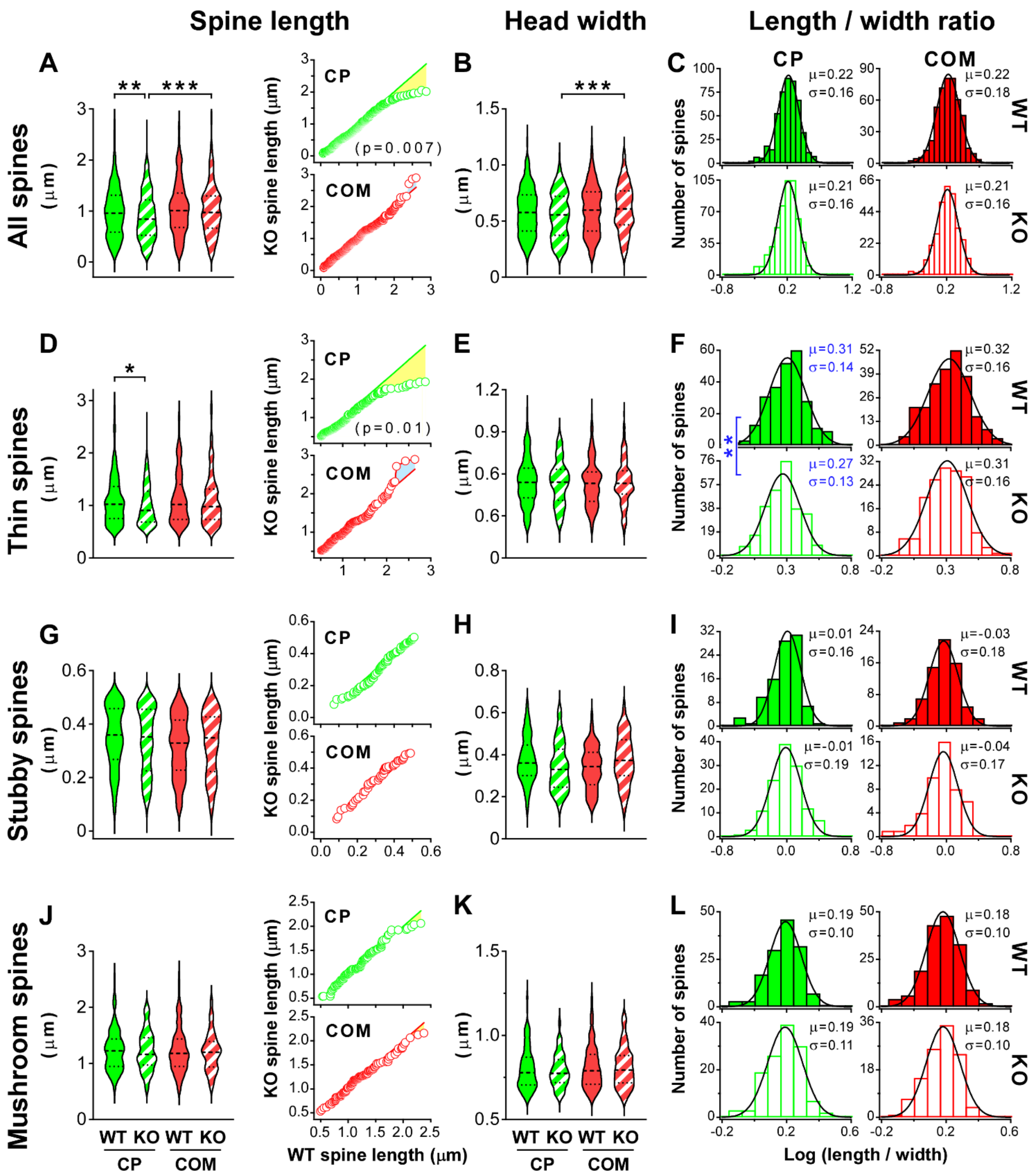

Supplemental Figure 2. Length and head width of basal dendritic spines in corticopontine and commissural layer V pyramidal neurons from WT and *Itgb3* KO mice. (A) Left, violin plot for the length of all dendritic spines (**p = 0.0043, ***p = 0.0004; non-parametric Kruskal-Wallis ANOVA followed by by Benjamini, Krieger and Yekutieli post-test, which corrects for multiple comparisons by controlling the false discovery rate). In each violin plot, the thick dotted line and the two thin dotted lines indicate the median and the quartiles, respectively. Right, all dendritic spines of CP *Itgb3* KO (top) or COM *Itgb3* KO

neurons (bottom) were ranked according to their length, resampled to match the number of dendritic spines of CP WT (top) or COM WT neurons (bottom) and plotted against the ranked dendritic spines of CP WT (top) or COM WT neurons (bottom). Straight green line: CP WT vs. CP WT; green open circles: CP *Itgb3* KO vs. CP WT; straight red line: COM WT vs. COM WT; red open circles: CP *Itgb3* KO vs. CP WT; p = 0.007; Kolmogorov-Smirnov test; n = 482, 532, 476 and 317 dendritic spines for CP WT, CP *Itgb3* KO, COM WT and COM *Itgb3* KO, respectively. **(B)** Violin plot for the head width of all dendritic spines (***p = 0.0002; non-parametric Kruskal-Wallis ANOVA followed by Benjamini, Krieger and Yekutieli post-test). **(C)** Histogram of the ratio between length and head width for all dendritic spines on a logarithmic scale (Log (length / width)). Continuous lines are Gaussian fits with the indicated means (μ) and standard deviations (σ). **(D-F)** As in (A-C) but for thin spines. Left of panel (D), top right of panel (D) and left of panel (F) show the same data as panels (F-H) of Fig 1. (*p = 0.01 in left panel of (D), Kruskal-Wallis ANOVA followed by Benjamini, Krieger and Yekutieli post-test; p = 0.01 in top right panel of (D), Kolmogorov-Smirnov test; **p = 0.004 in (F), Brown-Forsythe and Welch ANOVA followed by Benjamini, Krieger and Yekutieli post-test; n = 245, 276, 242 and 157 thin spines for CP WT, CP *Itgb3* KO, COM WT and COM *Itgb3* KO, respectively). **(G-I)** As in (A-C) but for stubby spines (n = 98, 127, 70 and 48 stubby spines for CP WT, CP *Itgb3* KO, COM WT and COM *Itgb3* KO, respectively). **(J-L)** As in (A-C) but for mushroom spines (n = 139, 129, 164 and 112 mushroom spines for CP WT, CP *Itgb3* KO, COM WT and COM *Itgb3* KO, respectively).

Materials and Methods

Animals

All experiments were performed in accordance with EU and Italian legislation. *Itgb3* KO mice (B6;129S2-Itgb3tm1Hyn/J, Jackson Laboratory) were described previously (1-4) and were backcrossed to the C57BL/6j background >10 times at the time of experiments.

Intracranial injections

Neurons projecting subcortically to the pons (corticopontine; CP) and intracortically to the contralateral cortex (commissural; COM) were labeled with EGFP by injecting the retrograde recombinant adeno-associated virus (retro-rAAV) AAVrg-hSyn-EGFP (1 µL; 1:5 dilution, titer: 1.5 x 10^{13} vg/mL, Cat. No. 50465-AAVrg, Addgene; (5)) into the pontine nuclei (A-P/M-L/D-V coordinates from Bregma: -4.16 / ±0.40 / 5.65 mm) and the contralateral prefrontal cortex (A-P/M-L/D-V coordinates from Bregma: 1.98 / + or -0.40 / 1.00 mm), respectively, at P28 (**Fig 1B**).

Immunohistochemistry

Ten to fifteen days post-infection, mice were intracardially perfused with 4% paraformaldehyde (PFA). The brain was postfixed for 6 hrs in 4% PFA at 4°C and cryoprotected in 30% sucrose; 50 µm-thick coronal sections of the prefrontal cortex were cut with a Sliding Microtome (HM 430, ThermoFisher scientific). Sections were permeabilized in 0.3% TritonX-100 for 30 min, blocked with 10% normal goat serum (NGS) for 1 hr and then incubated with a chicken anti-GFP primary antibody (1:1000; Cat. No. AB13970, Abcam) for 2 hrs and an Alexa Fluor488-conjugated anti-chicken secondary antibody (1:500; Cat. No. A11039, ThermoFisher scientific) for 1 hr.

Confocal microscopy and image analysis

Confocal stacks were acquired blind to the genotype with a Nikon Eclipse C1si using a 60x plan apochromat oil immersion objective (NA 1.40), 3x digital zoom, 0.96 µs pixel dwell time, 0.07 µm pixel size, 1 AU pinhole, 0.2 µm between optical sections and 3x scan averaging. Intensity of the 488 nm laser was set to 10 - 15% to achieve saturation of the somata and the PMT gain to 7 for all images.

We analyzed confocal images blind to the genotype using ImageJ. We filtered each stack using a Gaussian filter (radius: 0.5 pixels), Z-projected the maximal fluorescence intensities of in-focus stacks and applied the automatic ImageJ brightness/contrast. We analyzed dendritic spines in 17-31 regions of interests (ROIs), containing dendritic stretches of at least

10 µm long, from 3-4 different mice per condition. We excluded primary dendrites from the analysis and measured dendrite length across Z-stacks using the Neuroanatomy-SNT plugin of ImageJ (https://imagej.net/plugins/snt). In **Fig 1C**, intensity profiles were calculated after straightening the dendrites with the plugin Straighten (https://imagej.net/plugins/straighten) of ImageJ. The plugin SpineJ (6) of ImageJ was used to automatically identify dendritic spines and extract geometric information about the spines, namely length of the full spine (spine length), length of the spine neck (neck length) and width of the spine head (head width). For spine classification, we operationally defined (i) stubby spines as those with spine length <510 nm, (ii) mushroom spines as those with spine length >510 nm, head width >645 nm and neck length / spine length ratio <0.5, and (iii) thin spines as those with spine length >510 nm and either head width <645 nm or neck length / spine length ratio >0.5 (**Fig S1E**). 3D rendering was performed in Imaris 7.4 (Oxford Instruments).

Statistical analysis

Statistical differences were assessed using the Chi-square test, the Kolmogorov-Smirnov test, the two-way analysis of variance (ANOVA), the Kruskal-Wallis ANOVA or the Brown-Forsythe and Welch ANOVA followed by the Benjamini, Krieger and Yekutieli post-test, as indicated (Prism 7; GraphPad Software).

References

1. Cingolani LA, Goda Y. Differential involvement of beta3 integrin in pre- and postsynaptic forms of adaptation to chronic activity deprivation. Neuron Glia Biol. 2008;4(3):179-87. 10.1017/S1740925X0999024X.
2. Cingolani LA, Thalhammer A, Yu LM, Catalano M, Ramos T, Colicos MA, et al. Activity-dependent regulation of synaptic AMPA receptor composition and abundance by beta3 integrins. Neuron. 2008;58(5):749-62. 10.1016/j.neuron.2008.04.011.
3. Jaudon F, Thalhammer A, Zentilin L, Cingolani LA. CRISPR-mediated activation of autism gene Itgb3 restores cortical network excitability via mGluR5 signaling. Mol Ther Nucleic Acids. 2022;29:462-80. 10.1016/j.omtn.2022.07.013.
4. McGeachie AB, Skrzypiec AE, Cingolani LA, Letellier M, Pawlak R, Goda Y. beta3 integrin is dispensable for conditioned fear and Hebbian forms of plasticity in the hippocampus. Eur J Neurosci. 2012;36(4):2461-9. 10.1111/j.1460-9568.2012.08163.x.
5. Tervo DG, Hwang BY, Viswanathan S, Gaj T, Lavzin M, Ritola KD, et al. A Designer AAV Variant Permits Efficient Retrograde Access to Projection Neurons. Neuron. 2016;92(2):372-82. 10.1016/j.neuron.2016.09.021.
6. Levet F, Tønnesen J, Nägerl UV, Sibarita JB. SpineJ: A software tool for quantitative analysis of nanoscale spine morphology. Methods. 2020;174:49-55. 10.1016/j.ymeth.2020.01.020.
